# Adult-Onset Obesity Reveals Prenatal Programming of Glucose-Insulin Sensitivity in Male Sheep Nutrient Restricted during Late Gestation

**DOI:** 10.1371/journal.pone.0007393

**Published:** 2009-10-14

**Authors:** Philip Rhodes, Jim Craigon, Clint Gray, Stuart M. Rhind, Paul T. Loughna, David S. Gardner

**Affiliations:** 1 School of Veterinary Medicine and Science, University of Nottingham, Sutton Bonington, United Kingdom; 2 School of Biosciences, University of Nottingham, Sutton Bonington, United Kingdom; 3 Macaulay Land Use Research Institute, Craigiebuckler, Aberdeen, United Kingdom; Institute of Preventive Medicine, Denmark

## Abstract

**Background:**

Obesity invokes a range of metabolic disturbances, but the transition from a poor to excessive nutritional environment may exacerbate adult metabolic dysfunction. The current study investigated global maternal nutrient restriction during early or late gestation on glucose tolerance and insulin sensitivity in the adult offspring when lean and obese.

**Methods/Principal Findings:**

Pregnant sheep received adequate (1.0M; CE, *n* = 6) or energy restricted (0.7M) diet during early (1–65 days; LEE, *n* = 6) or late (65–128 days; LEL, *n* = 7) gestation (term ∼147 days). Subsequent offspring remained on pasture until 1.5 years when all received glucose and insulin tolerance tests (GTT & ITT) and body composition determination by dual energy x-ray absorptiometry (DXA). All animals were then exposed to an obesogenic environment for 6–7 months and all protocols repeated. Prenatal dietary treatment had no effect on birth weight or on metabolic endpoints when animals were ‘lean’ (1.5 years). Obesity revealed generalised metabolic ‘inflexibility’ and insulin resistance; characterised by blunted excursions of plasma NEFA and increased insulin_AUC_ (from 133 to 341 [s.e.d. 26] ng.ml^−1^.120 mins) during a GTT, respectively. For LEL *vs.* CE, the peak in plasma insulin when obese was greater (7.8 *vs.* 4.7 [s.e.d. 1.1] ng.ml^−1^) and was exacerbated by offspring sex (i.e. 9.8 *vs.* 4.4 [s.e.d. 1.16] ng.ml^−1^; LEL male *vs.* CE male, respectively). Acquisition of obesity also significantly influenced the plasma lipid and protein profile to suggest, overall, greater net lipogenesis and reduced protein metabolism.

**Conclusions:**

This study indicates generalised metabolic dysfunction with adult-onset obesity which also exacerbates and ‘reveals’ programming of glucose-insulin sensitivity in male offspring prenatally exposed to maternal undernutrition during late gestation. Taken together, the data suggest that metabolic function appears little compromised in young prenatally ‘programmed’ animals so long as weight is adequately controlled. Nutritional excess in adulthood exacerbates any programmed phenotype, indicating greater vigilance over weight control is required for those individuals exposed to nutritional thrift during gestation.

## Introduction

It is estimated that, at present, every 3–4 out of 10 adults worldwide are obese,defined as a body mass index of ≥30 kg/m^2^ (International Obesity Taskforce; http://www.iotf.org/database/index.asp) which represents a huge economic burden [1]. The early (fetal and neonatal) developmental environment has been proposed as a key sensitive period for increasing susceptibility to adult-onset obesity [2]. Programming of body composition and non-communicable disease (for which obesity is a significant risk factor) has been identified by the Grand Challenges Global Partnership as a research priority in order to mitigate the rising rise of diabetes and cardiovascular disease [3].

To date, several laboratory animal concept studies have shown proof-of-principle with regard to the developmental ‘programming’ of adult health, for review see [4], [5]. Most of these studies have used the laboratory rodent in which marked reductions or increases in macronutrient provision during gestation may or may not reduce birth weight, and when coupled with marked hypercaloric nutrition postnatally e.g. of fat or simple sugars, then a ‘programmed’ disadvantageous phenotype emerges [6], [7]. It is important, however, when extrapolating such interesting results to the human that the marked differences in energy partitioning, allometry, metabolic rate and life history traits between laboratory and larger animals are taken into account [8]–[10]. Therefore, to conduct similar nutritional and metabolic studies in larger animal models is imperative and to date the sheep and pig has been widely used.

In pigs, it has been shown that reduced birth weight enhances fat deposition which impacts upon glucose tolerance in these animals [11]. In the sheep, historically used as the animal model of choice for fetal developmental physiological studies because of the similarities in fetal physiology, endocrinological maturity and weight at birth to the human fetus [4], prenatal undernutrition has been shown to have a number of physiological consequences on the adult offspring dependent upon the stage of gestation the nutritional insult occurs [12], [13]. However, in these large animal studies the experimental endpoint has invariably been to characterise a given phenotypic consequence, and have been conducted when the animals have been raised in an environment appropriate for that species (e.g. for sheep, on pasture for 1–3 years; [14], [15]). Thus, invariably, at the time of study these animals are relatively lean and are physically active on a daily basis making the results of limited applicability to the current human condition in the Western World. In addition, given the increased ‘metabolic reserve’ of larger animals i.e. their greater ability to accommodate periods of undernutrition and their relatively slower metabolism, compared with laboratory rodents [8], it is all the more remarkable that developmental programming has been shown at all in larger animals. Clearly, therefore, early environmental ‘metabolic imprinting’ of a thrifty phenotype, i.e. after maternal undernutrition [16] has the potential to have an enormous impact in the context of the nutrition transition in the Western world [17]. In an experimental context, it is important to challenge the nutrition-transition paradigm i.e. the interaction between the pre- and postnatal nutritional environments, as has been done often in laboratory studies [6], [7], but much less so in larger animal work.

Consequently, we have previously developed a model of juvenile-onset obesity after prenatal undernutrition in sheep to test the predicted low-high/thin-fat deleterious phenotype. We find, as expected, that juvenile-onset obesity *per se* is metabolically disadvantageous but the pre-postnatal nutritional interactions are not always so clear cut or indeed evident [18]–[20]. To the best of our knowledge, no study to date in larger animal models has directly tested an interaction between prenatal undernutrition followed by a regime of postnatal nutritional excess adopted as an adult. Adult obesity is known to influence the function of multiple organs, encapsulated in the term ‘metabolic inflexibility’ [21] in which animals have a reduced ability to switch substrate-level oxidation between carbohydrate and lipid sources [22]. Epidemiological evidence from the Dutch Hunger Winter Famine suggests that specific periods of gestation may impact upon different organ systems in adult life to induce a metabolic phenotype that leads to obesity [23]. Thus, in this study, we aim to test the hypotheses that prenatal global nutrient restriction in sheep will interact with postnatal obesity to influence metabolic flexibility in the adult offspring and the response will be dependent on the gestational stage at which the nutritional insult occurred. Metabolic flexibility will be assessed in the lean and obese state (body composition determined by dual-energy X-ray absorptiometry) by measurement of carbohydrate, fat and the protein metabolite response to a glucose tolerance test.

## Results

### Weight at birth, postnatal growth and body composition at 1.5 years of age

There was no treatment or gender effect on birth weight, but a significant effect of fetal number (*P* = 0.004), with twins being born lighter than singletons (4.1 kg±0.2 [n = 12] *vs.* 5.3 kg±0.2 [n = 8], respectively). Postnatal growth to weaning was unaffected by treatment or fetal number but males grew at a faster rate (*P* = 0.02) than females (394±16 vs. 341±16 g.day^−1^, respectively) so that males were significantly larger than females at weaning (32.4±1.2 *vs.* 28.3±1.2 kg; *P*<0.01) and at 1.5 years (67.2±1.2 *vs.* 52.6±1.2 kg; *P*<0.01). Thereafter, whilst fetal number was retained as a potential explanatory variable in all analyses, it did not significantly influence any outcome measure. Thus, where appropriate in the data, only treatment and gender differences and any potential interactions are highlighted. At 1.5 years of age and ‘lean’, there was no effect of prenatal diet on body composition (Table 1). However, there were clear effects of gender; males had higher % body fat (17.48 *vs.* 13.54%, s.e.d. 1.60) and greater absolute fat mass, (9.88 *vs.* 6.27 kg, s.e.d. 0.98), lean mass (46.53 *vs.* 40.02 kg s.e.d. 2.54) and bone mineral content (1400 *vs.* 1170 kg s.e.d. 60) than females (Table 1).

**Table 1:** Body composition of prenatally undernourished sheep when lean at 1.5 years of age.

		CE	LEE	LEL	s.e.d	*T*	*G*	T*G
Fat (%)	male	16.93	15.95	19.57	2.76	NS	0.02	NS
	female	13.75	13.67	13.20	″	″	″	″
Fat mass (kg)	male	8.94	9.17	11.54	1.69	NS	0.01	NS
	female	5.97	6.65	6.18	″	″	″	″
Lean mass (kg)	male	44.31	48.11	47.18	4.39	NS	0.02	NS
	female	37.57	42.37	40.12	″	″	″	″
BMD (g.kg^3^)	male	1.10	1.12	1.11	0.03	NS	NS	NS
	female	1.09	1.06	1.09	″	″	″	″
BMC (g)	male	1368	1398	1436	118	NS	0.01	NS
	female	1121	1211	1193	″	″	″	″

Data are predicted means with the average standard error of the difference (s.e.d with 14 degrees of freedom in all cases) for the comparison. CE, Controls (n = 6); LEE, Low Energy Early (n = 7), LEL, Low Ener

gy Late (n = 7). Body composition were analysed by DXA (see Methods). BMD- Bone Mineral Density. BMC- Bone Mineral Content. T, main effect of treatment; G, main effect of gender; T*G, interaction between treatment and gender.

### Effect of an obesogenic environment on body composition and appetite

At 2 years of age, whilst the obesogenic environment facilitated considerable weight gain in all animals (Figure S2), body composition remained similar between treatment groups (Table 2). However, the previously observed gender effects remained; males had higher % body fat (32.67 *vs.* 30.27%, s.e.d. 1.85) and greater absolute fat mass, (21.38 *vs.* 16.70 kg, s.e.d. 1.48), lean mass (44.06 *vs.* 38.32 kg s.e.d. 2.1.76) and bone mineral content (1746 *vs.* 1500 g s.e.d. 72) than females (Table 1). When considering the change in weight with time, all measured variables significantly increased (*P*<0.001 all cases) except lean body mass which significantly decreased. For most comparisons there were no significant time*treatment or time*treatment*gender interactions for any aspect of the change in body composition after prolonged exposure to an obesogenic environment (i.e. Table 1 *vs.* Table 2). However, some effects were noted; there was a trend (P = 0.08) for LEL to put on less fat mass (effect size, -1616 g s.e.d. 1189) and the increase in BMD was greater (P<0.05) in LEE females (effect size, 0.12 s.e.d. 0.03) *vs.* control groups. After the period of weight gain, body weight and thus composition was stabilised throughout the experimental studies. At this time of weight stabilisation, individual food intake (over 24 h) and appetite (over 2 h) were assessed. There was no effect of treatment or gender on daily food intake (CE, 20.0; LEE, 21.3; LEL, 19.2 MJ/day s.e.d. 1.80) or appetite (i.e. 2 h intake for CE, 13.6; LEE, 13.3; LEL, 13.8 MJ/day s.e.d. 1.73).

**Table 2:** Body composition of prenatally undernourished sheep when obese at 2 years of age.

		CE	LEE	LEL	s.e.d	*T*	*G*	T*G
Fat (%)	male	31.73	32.02	34.27	3.19	NS	NS	NS
	female	32.95	29.93	27.93	″	″	″	″
Fat mass (kg)	male	21.16	20.82	22.15	2.55	NS	0.01	NS
	female	17.05	17.66	15.40	″	″	″	″
Lean mass (kg)	male	45.42	44.15	42.60	3.04	NS	0.09	NS
	female	34.77	41.11	39.05	″	″	″	″
BMD (g.kg^3^)	male	1.21	1.14	1.14	0.03	0.09	NS	NS
	female	1.15	1.16	1.13	″	″	″	″
BMC (g)	male	1832	1710	1695	124	NS	0.01	NS
	female	1450	1599	1450	″	″	″	″

Data are predicted means with the average standard error of the difference (s.e.d with 14 degrees of freedom in all cases) for the comparison. CE, Controls (n = 6); LEE, Low Energy Early (n = 7), LEL, Low Energy Late (n = 7). Body composition were analysed by DXA (see Methods). BMD- Bone Mineral Density. BMC- Bone Mineral Content. T, main effect of treatment; G, main effect of gender; T*G, interaction between treatment and gender.

### Effects of prenatal nutrient restriction and postnatal obesity on glucose tolerance and insulin sensitivity

#### Glucose tolerance

When lean, fasted plasma glucose concentrations were similar between dietary groups (CE, 3.65; LEE, 3.95; LEL, 3.70 mmol.L^−1^ s.e.d. 0.49) and gender (male, 3.91 *vs.* female, 3.61 mmol.L^−1^ s.e.d. 0.40). Similarly, fasted plasma insulin concentrations were not different between dietary groups (CE, 0.29; LEE, 0.37; LEL, 0.35 ng.ml^−1^ s.e.d. 0.09) or gender (male, 0.30 vs. 0.38 ng.ml^−1^ s.e.d. 0.07). On becoming overweight, fasted plasma glucose concentrations significantly reduced (P<0.01) relative to when lean but remained similar between dietary groups (CE, 3.28; LEE, 3.29; LEL, 3.08 mmol.L^−1^ s.e.d. 0.17) and gender (male, 3.12 *vs.* female, 3.31 mmol.L^−1^ s.e.d. 0.14). Fasted plasma insulin when obese remained at a similar level as when lean (CE, 0.38; LEE, 0.42; LEL, 0.43 mmol.L^−1^ s.e.d. 0.09) and was similar between sexes (male, 0.35 *vs.* female, 0.47 mmol.L^−1^ s.e.d. 0.07).

**Figure 1:**
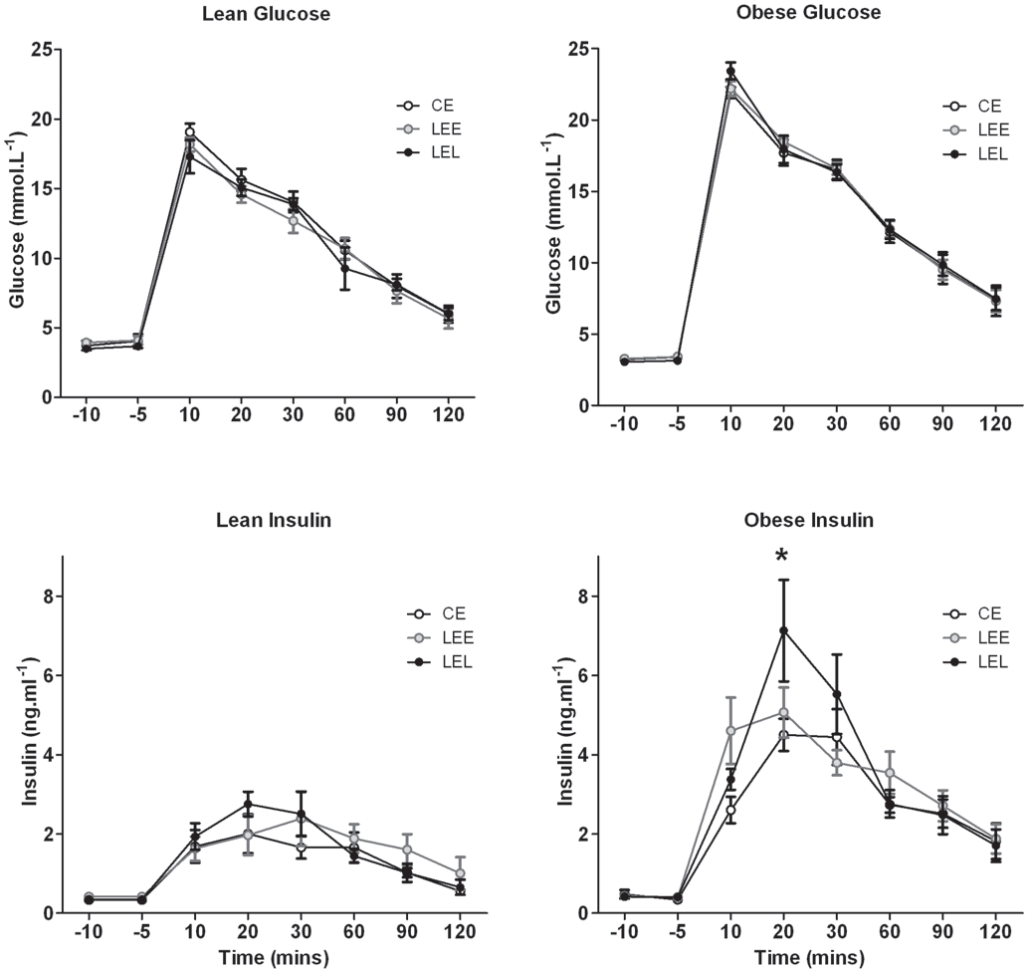
The response of prenatally nutrient restricted adult sheep to a glucose tolerance test when lean and obese. Data are Mean ± SEM. Glucose was administered I.V. (0.5 g.kg^−1^) and blood samples collected for measurement of plasma glucose and insulin (by ELISA, see Methods). Statistics are *, P<0.05 LEL *vs.* CE and LEE for peak insulin concentration.

**Figure 2:**
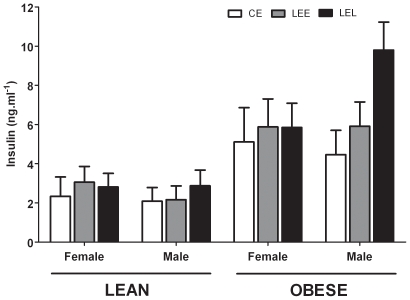
Peak plasma insulin in lean and obese male and female prenatally nutrient restricted adult sheep during a glucose tolerance test. Data are predicted means and SEM. CE, Controls (n = 6); LEE, Low Energy Early (n = 7), LEL, Low Energy Late (n = 7). Data were analysed by repeated measures general linear model revealing a significant effect of time (*; P<0.001 all cases) or a significant interaction (P<0.05) between time*treatment*gender (†).

Administration of I.V. glucose induced a significant rise in plasma [glucose] followed, ∼15–20 mins later, by a significant increment in plasma [insulin] (Figure 1). There were no treatment or gender related effects on glucose tolerance (area under the glucose response curve) or insulin sensitivity (area under the insulin response curve) when the animals were lean. Obesity, however, revealed a significant main effect of time and a time*treatment*gender interaction (P = 0.04) upon insulin sensitivity (Figure 1); both the glucose and insulin response curves were significantly greater relative to when lean (Figure 1), but the peak plasma insulin achieved was significantly greater in male LEL relative to both CE and LEE males (Figure 2).

#### Insulin sensitivity

**Figure 3:**
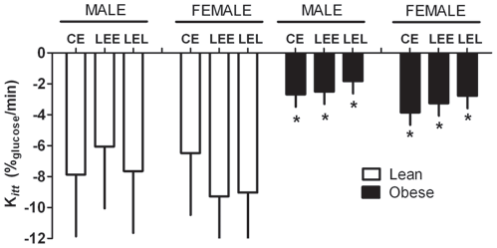
Insulin sensitivity of male and female lean or obese adult sheep after an IV ITT test (A). Data are predicted mean with the average s.e.d. for each comparison. Insulin (0.25 IU/kg^−1^ when lean or 0.75 IU.kg^−1^ when obese) was injected I.V. at time zero. Statistics are *, P<0.0001, relative to lean.

Injection of insulin reduced plasma glucose by 0.92 (s.e.d. 0.08) when lean and 1.12 (s.e.d. 0.13) mmol.L^−1^ when obese. The K_itt_ was markedly influenced by obesity but not by treatment group or gender (Figure 3). The response was only influenced by sex (P = 0.019) with males, regardless of time, having higher plasma glucose than females (by on average 0.39 (s.e.d. 0.14) mmol.L^−1^). This effect was greater still when adjusted for body weight (0.65 (s.e.d. 0.24 mmol.L^−1^).

### Effects of prenatal nutrient restriction on fat metabolism

#### Baseline status

Consideration of resting fat metabolism indicated no main effects of treatment or gender or any significant interactions with time (i.e. lean to obese). An exception was that basal plasma glycerol was lower in LEL and plasma cholesterol in LEE vs. other groups (Table S1). As expected, becoming obese significantly increased the resting plasma concentration of triglyceride, cholesterol, HDL and leptin relative to lean animals (Table S1) but, in sheep, led to a decrease in plasma NEFA and glycerol. Plasma LDL remained unchanged with increased obesity (Table S1).

#### Change during the GTT

Following injection of glucose, plasma NEFA, TAG and glycerol concentrations declined (P<0.01) in both lean and obese animals. These indices of lipid metabolism in response to high plasma glucose and insulin concentration during the GTT were unaffected by treatment group, but were sex-specific (Figure S3). The area under the response curve for plasma NEFAs was significantly lower in the obese relative to lean state in both males and females but, for triglyceride, the AUC was significantly greater in obese females relative to all other groups (Figure S3).

#### Change during the ITT

When a bolus dose of insulin was injected during euglycaemia, plasma NEFA increased immediately and was sustained (P<0.05), whereas plasma TAG only increased transiently (Figure S4). For these responses there were no significant treatment or gender interactions. Plasma glycerol, measured at three time points during the ITT (0, 8 and 16 mins), matched the NEFA profile i.e. a sustained increase was observed (data not shown).

### Effects of prenatal nutrient restriction on protein metabolism

As an index of lean mass metabolism we measured the fasted plasma concentrations of amino acids and urea in the lean and obese state. In general, plasma amino acid concentrations and urea were significantly lower in the obese relative to the lean state, (8191 *vs.* 5289 (s.e.d. 639) nmoles.ml^−1^; urea 8.59 *vs.* 5.70 (s.e.d. 0.42) mmol.L^−1^), with no effect of prenatal diet or gender or any interactions on this response. Given the multiple levels of interaction between individual amino acids we employed a multivariate analysis tool, principal components analysis, to identify any components that may explain the majority of the variation in amino acids with obesity. Three principal components were identified that accounted for >95% of the variation in amino acid concentrations between groups from the lean to obese phenotype; PC1 accounted for 85.6% of the variation, PC2, 8.1% and PC3, 2.4%. For PC1, the greatest latent vector loadings (i.e. indicating those amino acids contributing most to the variation) were the reductions in the plasma concentration of glycine (0.9660), alanine (0.1437), valine (0.0887) and glutamic acid (0.0687) and the increases in the plasma concentration of serine (−0.1518). The respective changes in the absolute plasma concentration for these amino acids are given in Figure S4. Using the principal component scores for PC1, as independent variates in a general linear regression model indicated that the amino acid variation reflected by PC1 was significantly associated with the decline in plasma [urea] concentration. There was no effect of prenatal treatment or gender on these effects.

### Assessment of metabolic flexibility in the lean and obese state

#### Baseline

Fasted blood biochemistry when lean and obese are given in Table S2. With obesity, animals became more alkalotic as reflected in increases in blood pH, p*_v_*O_2_ and acid-base excess and a decrease in *P_v_*CO_2._ In addition, blood electrolytes also changed significantly; Ca^2+^ increased, whilst Na^+^ and K^+^ declined (Table S2).

#### During the GTT

Changes in metabolic flexibility were indicated from a comparison of the delta change in lipid metabolites during the lean or obese GTT, for example, the blunting of the increase in plasma NEFA (Figure 5S5). Therefore, we also considered the delta change (i.e. values at 120 mins minus values at 0 mins) in blood biochemistry to mark metabolic flexibility: again, we saw no effect of sex (data not shown) and little effect of prenatal diet (treatment) influencing this response (Table 3). However, the delta change in blood biochemistry during a GTT changed with obesity (i.e. a significant effect of time was indicated); the changes in pH, *P_v_*CO_2_, *P_v_*O_2_, ABE, were all blunted (Table 3). Haemoglobin (Hb) concentrations and haematocrit (Hct) were similar in all groups when measured as obese animals (Hb: CE, 14.0±0.7; LEE, 15.3±0.6; LEL, 12.9±0.6 g.dL^−1^; Hct: CE, 36.5±2.2; LEE, 36.4±1.9; LEL, 37.1±1.9 %).

**Table 3:** The delta change in metabolites during a GTT when lean or obese.

		CE	LEE	LEL	s.e.d	*T*	*G*	T*G
pH	lean	0.06	0.07	0.06	0.03	NS	0.01	NS
	obese	−0.01	0.02	0.01	″	″	″	″
*p*CO_2_	lean	−7.73	−5.58	6.00	4.31	NS	0.02	NS
(mmHg)	obese	0.45	−1.17	−1.17	″	″	″	″
*p*O_2_	lean	9.48	6.91	8.45	9.17	NS	0.02	NS
(mmHg)	obese	−14.65	−1.98	1.42	″	″	″	″
ABE	lean	0.36	2.30	1.90	1.32	NS	0.04	NS
(meQ.L^−1^)	obese	−1.35	1.60	−0.01	″	″	″	″
K^+^	lean	−0.46	−0.43	−0.22	124	NS	0.01	NS
(mmol.L^−1^)	obese	−0.30	−0.32	−0.12	″	″	″	″
Na^+^	lean	−1.00	−0.85	−1.85	1.13	0.06	NS	NS
(mmol.L^−1^)	obese	1.83	−1.42	−1.42	″	″	″	″
Ca^++^	lean	−0.02	−0.02	−0.01	0.02	NS	NS	NS
(mmol.L^−1^)	obese	−0.03	−0.04	−0.01	″	″	″	″
Cl^−^	lean	0.83	−0.85	0.00	1.22	NS	NS	NS
(mmol.L^−1^)	obese	−0.16	−0.57	−0.42	″	″	″	″

Data are predicted means with the average standard error of the difference (s.e.d with 14 degrees of freedom in all cases) for the comparison. CE, Controls (n = 6); LEE, Low Energy Early (n = 7), LEL, Low Energy Late (n = 7). T, main effect of treatment; Ti, main effect of time (i.e. becoming obese); T*Ti, interaction between treatment and time.

### Post Mortem data

There were no treatment effects on absolute or relative wet organ weights at 2 years of age. Absolute weights were greater in male relative to female offspring, as expected, but when expressed in relative terms, few effects were observed with the exception of the brain, which was significantly larger in females than in their male counterparts (Table S3).

## Discussion

We have shown that global nutrient restriction of the pregnant ewe from mid-late gestation leads specifically to a greater early-phase (first 20 mins) increase in plasma insulin in response to an intravenous glucose bolus in the adult offspring. The response was sex-specific (greater in males > females) and only observed when the adult offspring were obese. In addition, for this study we broadly assessed metabolic health in the adult offspring when lean and the deterioration in metabolic health when they became obese. Obesity was clearly detrimental to metabolic health in many respects (lipoprotein and cholesterol profile, insulin sensitivity) but, contrary to our hypothesis, we found little evidence of greater deterioration of health in those offspring prenatally undernourished by global energy deficit. Taken together, the current study provides evidence to suggest that prenatal energy deficit has a sex-specific effect on adult glucose-insulin sensitivity when, and only when, excess calories are consumed; if the offspring remain lean and physically active through adolescence and early adulthood then good metabolic health prevails. The extent to which metabolic deterioration in programmed offspring may by exacerbated by older age cannot be determined by this study but clearly, prevention of excess weight gain through controlled food intake and lots of habitual physical activity can be inferred.

Pregnant women that suffered the Dutch Hunger Winter Famine during WWII, with the severe nutrient deficiency that it entailed gave birth to babies that were on average only ∼300 g less than contemporaneous women that were un-exposed [24]. The middle-aged adult offspring, however, had elevated plasma lipids, obesity and an increased incidence of coronary heart disease if exposed during early gestation [25], whereas those exposed during late gestation had reduced glucose tolerance [23]. The current study broadly concurs with these epidemiological observations and suggests the liver as the likely candidate for reduced whole body glucose tolerance (e.g. blunted first pass insulin metabolism and insulin-induced suppression of hepatic glucose output). This is inferred from our results indicating greater early (0–20 mins) plasma insulin concentrations in LEL (exposed to undernutrition during late gestation) relative to control animals in response to the GTT, but no difference in their response to an I.V. ITT (i.e. as a measure of insulin-dependent peripheral glucose uptake). In particular, we noted a clear treatment*gender interaction in the response to the GTT when obese, with male offspring mounting a much greater first phase insulin response relative to females. Given that no such interaction was observed during the ITT, as previously shown by Poore *et al*. [26], suggests that in offspring undernourished during late gestation the major sites of peripheral glucose uptake (skeletal muscle and adipose tissue) are not overtly resistant to insulin action *per se*. Rather, when hyperglycaemia and hyperinsulinaemia co-exist, such as postprandially (or, for example, after a GTT), differences in other insulin-sensitive tissues (pancreas and liver) that are not activated during the ITT but are during the GTT must account for the observed significant treatment*gender interaction.

In terms of the developmental programming paradigm the sex-specificity of many diverse programmed sequalae has been the source of much debate, but in general, it would appear that the male offspring are particularly vulnerable. For example, such a sex-specific effect on glucose-insulin metabolism has also been observed in the male offspring from mothers that were methyl-deficient around the time of conception [27] or nutritionally undernourished during gestation, in similarity to the current study [28]. Males also appear more susceptible with respect to the programming of function in alternative organs e.g. the heart and kidney [29], [30]. In the present study the males were castrates, therefore mitigating any effect of postnatal reproductive hormones. Thus, the sex-specific differences may relate to either female reproductive hormones [31], [32] or a delayed effect pertaining to sex-specific differences in the intrauterine response to nutrient deficit *per se* [33]. From the present results and taking into account other studies it is still not clear what mechanism may underlie the sex-specificity of developmental programming, although recent work would suggest there is differential sensitivity to epigenetic programming and altered methylation of key genes could underpin many of the sex-specific responses [27], [34]; the results, however, do emphasise the importance of including sex as a factor in the design of developmental programming experiments.

We have previously shown that late gestation undernutrition can marginally reduce glucose tolerance in *lean* adult sheep [14] in contrast to the current study, where reduced glucose only became apparent with significant obesity. The reason for this difference is likely due to the milder nutritional challenge employed over late gestation and birth in the current study (i.e. a 30% reduction in the current study *vs*. 50% in the previous study [14]). In addition, it is important to reiterate that ewes in the current study were also gradually realimented from day 128 to term to avoid any potential confounding by carry-over effects of prenatal undernutrition into lactation [35]. This can be considerable and likely affects many previous studies in this area; in the current study, that singles and twins grew at equivalent rates during lactation suggests that the lactational ability of ewes in our study was not hampered by prenatal undernutrition. Our study therefore uniquely illustrates that isolated mid-late gestation global undernutrition can have specific long-term effects on glucose-insulin dynamics in the adult male offspring when they become overweight.

In the ruminant in the resting state (euglycaemia), glucose clearance is likely orchestrated by Glut4-mediated insulin-dependent transfer into skeletal muscle and adipose tissue. During hyperglycaemia, clearance is additionally coupled to mass-action passive glucose transfer into pancreatic and hepatic cells (via Glut2, which has a high K_m_ for glucose; [36]). Thus, in contrast to our previous observation in which a reduction in adipose Glut4 was associated with the decline in insulin sensitivity [14], in the current study it would appear that the key ‘programmed’ effect is on either hepatic insulin resistance (i.e. decreased first-pass hepatic insulin extraction, which is approximately 50% in the sheep under basal conditions, [37] or insensitivity to insulin-induced blunting of hepatic glucose output) and/or pancreatic dysfunction (e.g. insulin hypersecretion, [38]). As yet, we are unable to discern a pancreatic from hepatic effect, although the available evidence from other animal models that have attempted to address this conundrum suggest a combination of both [39]. Certainly, it would appear that at a systemic level potential changes in skeletal muscle fibre type [13] and/or skeletal muscle insulin signalling protein expression [40] are not important in mediating the differences in early insulin secretion in the current study.

From literature in Man and in animal models it is apparent that following a deficit in growth, rapid weight loss or undernutrition there is a period of preferential fat accretion or ‘catch-up fat’ [41]. This paradigm is akin to the original ‘thrifty phenotype’ hypothesis [16], the later ‘mis-match’ [42] or alternative thin-fat [17] hypotheses. Here, nutritional insufficiency leading to deficits in weight and/or growth (particularly during the key developmental periods of gestation and lactation) when coupled with postnatal nutritional excess increase the risk of adult metabolic disease [43]–[45] and actually reduce lifespan in male mice [7]. Hence, in the current study we specifically designed the experiments, using a relevant large animal model, to test this hypothesis. Our results largely agree with the null hypothesis in that few time*treatment interactions were observed; prenatal undernutrition did not make the adults more susceptible to greater adipose tissue gain *per se*, indeed, LEL put on significantly less fat than controls when exposed to an obesogenic environment. There are a number of reasons that may go some way to explaining this variance with the prevailing literature. First, when measured in a lean condition the animals had been in their habitual environment for 1.5 years, were relatively active and eating a low energy dense diet, in stark contrast to laboratory species [46] and much epidemiological data in Man. The animals were still relatively young (1.5 years) when sheep may live up to 12 years, however, catch-up fat has been observed from a young age [41]. Alternatively, 30% global nutrient restriction of the pregnant sheep could be considered a relatively milder challenge when compared to, for example, 50% protein restriction in the rat [4], [5]. The long-term consequences of prenatal programming may therefore be relatively harder to elucidate in larger animal models in comparison to small because of species-specific allometry and the metabolic ‘buffer’ offered by the larger body surface area:volume ratio of the sheep [12]. Nevertheless, with time, common programmed end-points have been shown to develop in larger animal species [15], [47], [48].

In this study we uniquely measured the development of obesity in sheep by repeated DXA which has a very low internal coefficient of variation and is very accurate for tissue weights from 500 g to 90 kg. However, we sought to further assess DXA against chemically determined body composition. Whilst there was good correlation between the two methods, chemical extraction of tissue for fat increased the apparent % fat determined by 75%. This is likely due to the Soxhlet method extracting absolutely all lipids available in that tissue (extracellular, intracellular, membraneous) rather than those that are simply metabolically available. Thus, the Soxhlet method is useful for determining fat composition of food to be eaten (as all fat will be extracted intestinally) but is perhaps less relevant in metabolic studies whereas DXA is a more appropriate method to determine body composition for assessing effects of adiposity on metabolism *in vivo*. Indeed, the value of 30% whole body fat mass in the current study determined by DXA concur with levels achieved previously by us after juvenile-onset obesity [19]. Adult onset obesity in the current study was associated with a significant decrement of lean mass (∼2–3 kgs), contrary to the often-reported increase with obesity, presumably as a consequence of increased weight-bearing [49]. Previous work in sheep, however, has also reported a reduction in carcass protein content with adult-onset obesity induced by overconsumption of feed [50]. The similar responses observed in that study and the current one, together with the reductions in plasma amino acid and urea concentrations, suggest reduced protein turnover in obese sheep, perhaps due to the reduced activity of the sheep within our ‘obesogenic’ environment. In Man, increased protein catabolism has been observed with obesity, related to the resistance of glucose and protein metabolism to insulin and the increased hepatic supply of glucogenic amino acids [51]. However, the fact that no overt peripheral insulin insensitivity was observed and the majority of the change in plasma amino acids could be attributed to only those few amino acids originally present in the highest concentrations in the plasma suggests, by the law of parsimony, that the increased dietary fat incorporation in the current study (from 3 to 6%) reduced rumen microflora protein synthesis and thus overall amino acid availability; the net effect being reduced protein turnover, and reduced measured amino acid and urea concentrations. Taken together with the changes in fat metabolism it would appear that obesity in sheep engendered by 7–8 months exposure to an ‘obesogenic environment’ created by increasing food and fat intake and reducing physical activity has put the sheep into a state of decreased protein turnover (likely through reduced protein synthesis) with net lipid deposition and decreased oxidation (as reflected in reduced plasma NEFA and glycerol) [50].

In addition, obesity in sheep was characterised by a decline in the individuals' capacity to transition between metabolic substrates under conditions of rapidly changing fuel supply, here recreated after a glucose tolerance test. The obese individual, in response to elevated blood glucose, responded with markedly blunted excursions in plasma metabolites and biochemistry (Table 3 for plasma biochemistry and Supplementary Figure S5 for NEFA). Such a reduction in ‘metabolic flexibility’, for example a reduced transition from fatty acid efflux to storage in response to a carbohydrate rich meal [21] has recently been characterised in obese individuals and is thought to contribute to Metabolic Syndrome. We observed no overall effect of the prenatal diet on this response. However, for LEL, that had a greater plasma insulin response to high glucose coupled with reduced metabolic flexibility from being obese, then over time (i.e. longer exposure to the obesogenic environment), overt peripheral insulin resistance would perhaps be more likely to develop and be more pronounced in those sheep undernourished during late gestation [52].

In our study, there is potential that the effect is confounded by the study design in two ways; first, the increase in weight with obesity necessitated an increase in delivered glucose (administered v/w) relative to the first or lean GTT, therefore precipitating an overall greater response when obese. In defence, we highlight the fact that the gain in body weight was equivalent between treatment groups and that statistical adjustment of the insulin response to GTT for the weight gain (included as a co-variate) and thus the increased delivery of glucose, actually increased the F-statistic for the treatment*gender interaction, indicating a specific effect of the mismatch between pre- and post-natal diet on first phase insulin response in male offspring. Secondly, we acknowledge that following prior consultation with a statistician our best option for testing the hypothesis that prenatal undernutrition interacts deleteriously with postnatal nutritional excess was to use a repeated measures design. Such a design offers greater precision in testing for potential time*treatment*gender interactions as within animal variation is generally less than between animal variation. Furthermore, if no interactions are present (e.g. with time) then the study can more precisely determine potential main effects as the second measurement acts as a replicate of the first (i.e. the benefits of a factorial design); such experimental designs are therefore encouraged by the National Centre for the Reduction, Refinement and/or Replacement of animals in research (NC3Rs) [53]. Nevertheless, we are unable to control for an overall effect of time (being confounded with acquisition of obesity). However, we suggest that a 6-month period in an adult animal that may live for up to 12–15 years is not a significant length of time and that a doubling of fat-mass over that period is by far the over-riding effect. In addition, all treatment groups were randomly group housed together over this period, thus any effect of time *per se* would be expected to act equally on each individual sheep; given that our longitudinal design increased our sensitivity to observe time*treatment*gender interactions and we only observed such an effect of time (i.e. obesity) in one treatment*gender combination (male LEL) suggests that male LEL specifically differ with respect to groups in their response to adult-onset obesity.

In conclusion, global nutrient restriction of sheep had little delayed ‘programming’ effect on carbohydrate, fat and/or protein metabolism in the adult offspring when they were lean and physically active. However, when coupled with an obesogenic environment, those male sheep that experienced a poor diet during late gestation exhibited evidence of reduced insulin sensitivity.

## Materials and Methods

### Animals, ethical review and experimental design

All procedures were performed in accordance with the UK Animals (Scientific Procedures) Act, 1986 and were approved by the relevant local ethical review committees of the Macaulay Institute and the University of Nottingham.

Twenty Scottish Blackface, multiparous, ewes were synchronised in oestrus using progestagen sponges (Cronolone, 30 mg; Intervet, Cambridge, UK) and naturally mated with Scottish Blackface rams. Within 12 h of mating (designated as day 0 of gestation) ewes were individually housed indoors and offered either a complete diet (comprising a pelleted ration together with a fixed amount of hay (250 g, 1.7MJ)), providing adequate energy, micronutrient and vitamin and mineral requirements for liveweight maintenance (1.0x maintenance [M]) to term (∼147 days, Control Energy [CE], n = 6 offspring) according to the Agricultural & Food Research Council [54], or were fed a global nutrient-restricted diet (0.7M) during either early (1 to 65 days; Low Energy Early [LEE], n = 7 offspring) or mid-late gestation (65 to 128 days; Low Energy Late [LEL], n = 7 offspring). At all other times during gestation ewes received a diet providing 1.0M. The nutrient restriction periods were chosen to correspond with periods of either no or partial fetal luteinising and follicle stimulating hormone production (i.e. none up to day 65 and some thereafter) but also, partially, with fetal muscle development, which is largely proliferative up to day 80 and differentiating (hypertrophic) thereafter [55], [56]. After scanning at day 70 of gestation, the amounts of pelleted feed offered were adjusted according to stage of gestation and fetal number [57].

All sheep lambed naturally and were put out to pasture from two weeks of age. The three treatment groups were approximately balanced for female:male offspring (CE, 2∶4; LEE, 3∶4; LEL, 4∶3) with males being castrated shortly after birth. Supplementary feed (0.6–1 kg complete diet/ewe/day) was offered until weaning at 12 weeks. Offspring remained at pasture until 1.5 years when, after Home Office approval and veterinary clearance, they were moved to the Sutton Bonington Campus, University of Nottingham for further study.

After being moved, all offspring were weighed and acclimatised at pasture for two weeks before being group housed in barn accommodation where they were offered a complete diet at maintenance requirement. The diet provided 140 g.kg^−1^ crude protein (from dried barley and wheat grains), 70 g.kg^−1^ fibre, 3% fat (palm kernel oil) and 12.7 MJ/kg metabolisable energy. After two weeks, sheep were weighed and a jugular catheter inserted under local anaesthesia (0.5 ml Lignocaine hydrochloride) and after 24 h fasting all sheep were subjected, on separate days, to 1) a glucose tolerance test (GTT), 2) an insulin tolerance test (ITT) and 3) body composition analysis by dual energy x-ray absorptiometry (DXA). At this stage sheep were considered ‘lean’ (average body condition score of 2.4, scale of 1 = emaciated, 5 = obese) [58]. Thereafter, we reared the sheep in an ‘obesogenic’ environment to model the current ‘Western’ cultural environment, i.e. increased incorporation of fat in the diet (from 3–6% oil; crude protein [140 g.kg^−1^], metabolisable energy density [13.3 MJ/kg]), a larger portion size (offered 150% maintenance over two periods) and reduced physical activity [barn *vs.* pasture grazing reduces activity in sheep by 50–75% [18]] to encourage weight gain (a 50% increase over a 6-month period; BCS, 4.5+). This represented a theoretical shift in BMI for the sheep from a normal to obese category (22.5 to 32.5 kg/m^2^). When obese, body composition was stabilised through careful monitoring of weight gain and adjustment of food intake and all previously conducted protocols were repeated (GTT, ITT and DXA).

### Experimental protocols

#### Glucose Tolerance Test

After 24 h fasting, blood (5 ml) was withdrawn into a K^+^EDTA blood tube at −10 and −5 min relative to injection of a bolus dose of glucose solution (I.V. 0.5 g.kg^−1^; time zero). Subsequent blood samples (2 ml) were collected at 10, 20, 30, 60, 90 and 120 minutes. Whole blood was centrifuged (800 *g* for 10 mins at 4°C) and plasma collected and frozen at −20°C for further analysis.

#### Insulin Tolerance Test

The ITT assesses whole body euglycaemic insulin-dependent glucose uptake and correlates closely with the hyperinsulinaemic–euglycaemic clamp technique [59]. The dose of insulin given is designed to reduce resting plasma glucose by ∼1–1.5 mM over a 15–16 min period and from this the percentage decline in blood glucose concentration per minute (%min^−1^) from 4 to 16 min relative to baseline (0 and 2 mins) is calculated [60]. For sheep in this study, the dose required was 0.25 IU/kg (Novorapid, UK) when lean and 0.75 IU/kg when obese. Blood samples (2 ml) were collected into K^+^EDTA blood tubes at 2, 4, 6, 8, 10, 12, 14 and 16 minutes later. Whole blood was centrifuged (800 *g* for 10 mins at 4°C) and plasma collected and frozen at −20°C for further analysis of plasma metabolites.

#### Body composition

Animals were sedated (i.m. injection of 1.5 mg.kg^−1^ Ketamine with 0.1 mg.kg^−1^ Xylazine) and scanned in a transverse position using a Lunar DPX-L (fast-detail whole body smartscan). The scan lasted ∼15 min after which animals were allowed to recover in a pen and returned to the barn. Regular phantom spine and internal validations showed the DXA to be >97% reproducible. In addition, in a separate experiment, DXA-determined fat and fat-free body composition was validated against whole carcass chemical analysis. Briefly, 14 half-carcasses were macerated (WolfKing macerator) and 250 g dried, homogenized and nitrogen content determined by a FlashEA1112 nitrogen analyzer (Thermo Scientific, UK). Percentage fat was determined by rapid soxhlet extraction using a Gerhardt Soxtherm (Wolflabs, York, UK). All procedures have been described previously in detail, [13]. There was a significant correlation between methods (Pearson correlation 0.92, P<0.001; Figure 1a), but the DXA had a lower limit of fat detection in the sheep (at 4–5% body fat) and estimated ∼75% less body fat than complete chemical extraction, as indicated by a Bland-Altman plot (Figure S1).

#### Food intake

During the experimental period, individual food intake (feed and hay [8.6 MJ.kg^−1^ DM]) was assessed over at least 3 consecutive days on which there were no other measurements. In addition, on a separate day, food was withdrawn from the animals for at least 12–18 h and appetite assessed by measurement of food intake over a 2-hour period when food was re-introduced, *ad libitum* [61]. After all experiments the sheep were humanely euthanised by electrocortical stunning and exsanguination. All major organs were rapidly excised and weighed and stored at −80°C.

### Assays of blood and plasma

#### Blood biochemistry

During each GTT, baseline (−10 min) and +120 min whole blood samples were auto-analysed for concentrations of glucose, K^+,^ Na^+^, Ca^2+^, Cl^−^ (mmol.L^−1^) pH, *p*CO_2_, *p*O_2_, and HCO_3_^−^ (mmHg; ABL805-FLEX, Radiometer, Crawley, UK), Subsequently, all GTT plasma samples were assayed for concentrations (mmol.L^−1^) of glucose, urea, triacylglycerides (TAG), glycerol (µmol.L^−1^) and non-esterified fatty acids (NEFAs) whilst baseline samples were also analysed for concentrations of amino acids (nmoles.ml^−1^), cholesterol, HDL, LDL, glycerol (all mmol.L^−1^ and by Randox RX Imola, Co Antrim, UK), haemoglobin (g/dl; OSM3, Radiometer, Crawley, UK) and haematocrit (%). The latter two measurements in whole blood were only measured in obese animals. Plasma obtained during the ITT was analysed for concentrations of glucose, NEFA, glycerol and TAG (all mmol.L^−1^).

#### Analysis of amino acids and hormone

Amino acids were isolated from plasma and derivatised using the EZ:Faast^™^amino acid kit (Phenomenex, Macclesfield, UK) as previously described [62]. The method permitted the analysis of 18 amino acids, excluding cysteine and arginine. Plasma insulin concentrations (ng.ml^−1^) were determined, in duplicate, for all plasma samples obtained during each GTT using an ovine specific insulin ELISA kit (Mercodia AB; Uppsala, Sweden). Two pooled plasma samples, of high and low insulin concentration, provided internal quality control. Plates were read at 450 nm (Labsystems Multiskan Ascent plate reader plus Ascent software 2.6). Intra and inter-assay coefficients of variation were <10% and <15%, respectively. Plasma leptin concentrations (ng.ml^−1^) were determined using a validated double-antibody RIA as previously reported [18], [63]. Samples were assayed in triplicate (200 µl) using a rabbit anti-ovine leptin primary antibody, iodinated ovine leptin and sheep anti-rabbit secondary antibody. The limit of leptin detection was 0·1 ng.ml^−1^ and the intra- assay coefficients of variation for the assay was typically <5%.

### Statistics

#### Univariate analysis

The glucose, insulin, TAG, glycerol and NEFA data from each GTT were collated and baselines and the areas under the response curves (AUC) calculated using the trapezoid rule (Graphpad Prism 5). The data assessing prenatal diet on offspring characteristics (i.e. when ‘lean’ or when ‘obese’) were analysed as a general linear model with treatment (CE, LEE, LEL), fetal number (single or twin) and gender (male, female) as fixed effects. Twin lambs were treated as independents as each was from a different mother, but since they failed to explain any of the variation in any result after weaning, were not included in any further statistical models. The effect of becoming obese superimposed on prenatal undernutrition was assessed as a General Linear Mixed Model with repeated measures (i.e. data when lean and obese). *A priori* contrasts of interest to be examined were specifically time*treatment or time*treatment*gender interactions. ITT data were analysed by univariate GLM. Data are presented as predicted means from the model with either standard error of the treatment mean (S.E.M) or standard error of the difference between comparisons, as appropriate, used to represent the variance. For GLMM statistical comparisons we had n = 20 animals and therefore our total degrees of freedom (*df*) was 19 with 14 residual *df* (*df*_treatment_,2; *df*_sex_,1; *df*_treatment*sex_,2); thus, the 95% confidence interval for probability testing is ±2.057 the s.e.d. for that comparison. Effects were deemed significant when *P*<0.05, but *P = *0.06–0.09 were also highlighted to indicate that the effects were close to the significance boundary. All data were analysed with Genstat (v12).

#### Multivariate analysis

Amino acid concentrations in baseline plasma were analysed before each GTT test. Given the complex, non-independent interrelationships between amino acid pathways, simple univariate RM-GLM analysis for individual amino acids was not suitable to describe the changes in plasma concentration of multiple amino acids with obesity in sheep. Therefore the multivariate analysis principal component (PC) procedure was used to describe the change in amino acid levels with obesity [62]. PC analysis indicated that most of the variation in amino acid concentrations between the lean and obese state could be explained by three principal components which explained >95% of the variation in amino acid concentrations. Scores were assigned for each of these components and were incorporated into a generalised linear regression model to identify whether the variation in amino acid concentration defined by each PC significantly explained the variation in the independent variate e.g. resting [urea]. The latent vector loadings (which indicate the relative importance of the individual amino acids to a particular PC) for any PC that contributed significantly to a linear model were examined to determine which amino acids were most influential in defining the PC. All PC analyses were conducted using Genstat v11.

## Supporting Information

Table S1 The effect of adult-onset obesity on lipid metabolites in sheep. Data are predicted means with the average standard error of the difference (s.e.d with 14 degrees of freedom in all cases) for the comparison. CE, Controls (n = 6); LEE, Low Energy Early (n = 7), LEL, Low Energy Late (n = 7). NEFA, non-esterified fatty acids, HDL - High density lipoprotein. LDL - Low density lipoprotein. T, main effect of treatment; Ti, main effect of time (i.e. becoming obese); T*Ti, interaction between treatment and time.
(0.05 MB DOC)

Table S2 Baseline blood biochemistry in lean and obese adult sheep. The change in resting blood biochemistry from a lean to obese state as measdured on an ABL-800Flex (Radiometer Ltd, UK). Data are Grand Means with standard error of the difference and the df for the comparison of 14. P, for effect of time (i.e. onset of obesity). ns, not significant.
(0.04 MB DOC)

Table S3 Post Mortem organ weights in male and female sheep. Body and organ wet weights of male (n=11) and female (n=9) offspring at post mortem. Data are Grand Means±SEM. Statistics are *, **, ***, P<0.05, P<0.01 or P<0.001 respectively for male vs. female. ns, not significant. †, relative adrenal and pituitary weight expressed as (g.kg−1) ×103.
(0.04 MB DOC)

Figure S1 A) Validation of DXA-determined vs. chemically determined fat mass in sheep with B) a representative Bland-Altman plot. A) 14 sheep of differing body composition as determined by DXA were euthanised and body composition determined by chemical analysis (see Methods for details). The two methods were significantly correlated (P<0.001, Pearson correlation; spline with 95% CI shown) with an equation for the line of y = 1.79x+3.51. B) The Bland Altman plot illustrates the lower limit of detection for DXA in the sheep (4–5% fat) and that DXA vs. chemical analysis estimates ∼75% less whole body fat.
(1.11 MB TIF)

Figure S2 Weight gain in sheep reared in an ‘obesogenic’ environment. Data are Means±SEM. Sheep were group housed in a barn and fed at 1.5M for a period of 6–7 months to achieve a specified weight gain (see Methods). Baseline studies were conducted when animals were designated as ‘lean’ and were repeated when ‘obese’. There were no differences in weight gain between treatment groups.
(0.89 MB TIF)

Figure S3 The lipid metabolite response to a glucose tolerance test in male and female lean and obese sheep. Data are Mean±SEM for areas under the glucose response curve (respective AUC units). Statistics are *, P<0.05, lean vs. obese or †, for a time*gender interaction.
(2.55 MB TIF)

Figure S4 Amino acids contributing most to the variation in concentration with adult-onset obesity. Data are Mean±SEM for amino acid concentrations. Statistics are *, P<0.01, lean vs. obese.
(0.81 MB TIF)

Figure S5 The glucose, NEFA and triglyceride response of prenatally nutrient restricted adult sheep to an insulin tolerance test when obese. Data are Mean±SEM. Insulin (0.75IU.kg-1) was injected I.V. at time zero.
(0.90 MB TIF)
