# Supplementary material for: Adult-Onset Obesity Reveals Prenatal Programming of Glucose-Insulin Sensitivity in Male Sheep Nutrient Restricted during Late Gestation
Source: PLoS One. 2009 Oct 14;4(10):e7393. doi: 10.1371/journal.pone.0007393 (PMC2756957; doi:10.1371/journal.pone.0007393)
Supplement: Table S1 — The effect of adult-onset obesity on lipid metabolites in sheep. Data are predicted means with the average standard error of the difference (s.e.d with 14 degrees of freedom in all cases) for the comparison. CE, Controls (n = 6); LEE, Low Energy Early (n = 7), LEL, Low Energy Late (n = 7). NEFA, non-esterified fatty acids, HDL - High density lipoprotein. LDL - Low density lipoprotein. T, main effect of treatment; Ti, main effect of time (i.e. becoming obese); T*Ti, interaction between treatment and time. (0.05 MB DOC) [file pone.0007393.s001.doc]

**Table S1. The effect of adult-onset obesity on lipid metabolites in sheep.**

|  |  | **Experimental Group** | | |  | **Statistics** | | |
| --- | --- | --- | --- | --- | --- | --- | --- | --- |
| Triglyceride (mmol.L^-1^) | ***Gender*** | **CE** | **LEE** | **LEL** | **s.e.d** | ***T*** | ***Ti*** | ***T*Ti*** |
|  | lean | 0.16 | 0.15 | 0.14 | 0.02 | NS | <0.001 | NS |
|  | obese | 0.22 | 0.23 | 0.22 |  |  |  |  |
| Glycerol (µmol.L^-1^) | lean | 110 | 88 | 69 | 10 | 0.04 | 0.006 | 0.03 |
|  | obese | 70 | 66 | 71 |  |  |  |  |
| NEFA  (mmol.L^-1^) | lean | 0.99 | 0.88 | 0.84 | 0.11 | NS | <0.001 | NS |
|  | obese | 0.73 | 0.65 | 0.65 |  |  |  |  |
| Total cholesterol (mmol.L^-1^) | lean | 1.44 | 1.33 | 1.42 | 0.21 | 0.04 | <0.001 | 0.08 |
|  | obese | 2.52 | 1.75 | 2.31 |  |  |  |  |
| HDL  (mmol.L^-1^) | lean | 0.66 | 0.63 | 0.66 | 0.14 | 0.08 | <0.001 | 0.09 |
|  | obese | 1.43 | 0.99 | 1.36 |  |  |  |  |
| LDL  (mmol.L^-1^) | lean | 0.25 | 0.23 | 0.23 | 0.04 | NS | NS | NS |
|  | obese | 0.31 | 0.21 | 0.25 |  |  |  |  |
| Leptin  (ng.ml^-1^) | lean | 4.10 | 3.90 | 4.13 | 2.08 | NS | <0.001 | NS |
|  | obese | 12.63 | 10.99 | 10.36 |  |  |  |  |

Data are predicted means with the average standard error of the difference (s.e.d with 14 degrees of freedom in all cases) for the comparison. CE, Controls (n=6); LEE, Low Energy Early (n=6), LEL, Low Energy Late (n=7). NEFA, non-esterified fatty acids, HDL – High density lipoprotein. LDL – Low density lipoprotein. T, main effect of treatment; Ti, main effect of time (i.e. becoming obese); T*Ti, interaction between treatment and time.
