# Supplementary material for: Adult-Onset Obesity Reveals Prenatal Programming of Glucose-Insulin Sensitivity in Male Sheep Nutrient Restricted during Late Gestation
Source: PLoS One. 2009 Oct 14;4(10):e7393. doi: 10.1371/journal.pone.0007393 (PMC2756957; doi:10.1371/journal.pone.0007393)
Supplement: Table S2 — Baseline blood biochemistry in lean and obese adult sheep. The change in resting blood biochemistry from a lean to obese state as measdured on an ABL-800Flex (Radiometer Ltd, UK). Data are Grand Means with standard error of the difference and the df for the comparison of 14. P, for effect of time (i.e. onset of obesity). ns, not significant. (0.04 MB DOC) [file pone.0007393.s002.doc]

**Table S2. Baseline blood biochemistry in lean and obese adult sheep.**

|  | Baseline concentration | | | Statistics | |
| --- | --- | --- | --- | --- | --- |
|  | Lean | Obese | | *s.e.d* | *P* |
| pH | 7.38 | | 7.43 | 0.01 | 0.07 |
| *P_v_*CO_2_ (mmHg) | 47.22 | | 41.71 | 1.93 | 0.01 |
| HCO^-^_3_ (mmol.L^-1^) | 25.38 | | 26.37 | 0.81 | ns |
| *P_v_*O_2_ (mmHg) | 35.31 | | 51.94 | 4.95 | 0.01 |
| ABE (mEq.L^-1^) | 2.47 | | 3.06 | 0.78 | ns |
| Na^+^ (mmol.L^-1^) | 143 | | 140 | 0.41 | 0.001 |
| K^+^ (mmol.L^-1^) | 4.26 | | 4.03 | 0.07 | 0.01 |
| Ca^2+^ (mmol.L^-1^) | 1.16 | | 1.21 | 0.01 | 0.008 |
| Cl^-^ (mmol.L^-1^) | 104 | | 104 | 0.9 | ns |

The change in resting blood biochemistry from a lean to obese state as measdured on an ABL-800Flex (Radiometer Ltd, UK). Data are Grand Means with standard error of the difference and the *df* for the comparison of 14. *P*, for effect of time (i.e. onset of obesity). ns, not significant.
