# Supplementary material for: Adult-Onset Obesity Reveals Prenatal Programming of Glucose-Insulin Sensitivity in Male Sheep Nutrient Restricted during Late Gestation
Source: PLoS One. 2009 Oct 14;4(10):e7393. doi: 10.1371/journal.pone.0007393 (PMC2756957; doi:10.1371/journal.pone.0007393)
Supplement: Table S3 — Post Mortem organ weights in male and female sheep. Body and organ wet weights of male (n=11) and female (n=9) offspring at post mortem. Data are Grand Means±SEM. Statistics are *, **, ***, P<0.05, P<0.01 or P<0.001 respectively for male vs. female. ns, not significant. †, relative adrenal and pituitary weight expressed as (g.kg−1) ×103. (0.04 MB DOC) [file pone.0007393.s003.doc]

**Table S3.** *Post Mortem* organ weights in male and female sheep.

|  | Absolute weights | | *P* | Relative weights (g/kg) | | *P* |
| --- | --- | --- | --- | --- | --- | --- |
|  | Male | Female |  | Male | Female |  |
| Body weight (kg) | 82.2±01.5 | 66.5±1.2 | *** | - | - | - |
| Brain (g) | 103±2 | 95±4 | ns | 1.26±0.03 | 1.44±0.06 | ** |
| ^†^Pituitary (g) | 0.81±0.12 | 0.65±0.08 | ns | 9.71±1.27 | 9.87±1.07 | ns |
| Perirenal Fat (kg) | 2.88±0.30 | 2.39±0.30 | ns | 33.5±2.7 | 37.1±2.8 | ns |
| Pericardial Fat (g) | 219±15 | 161±9 | * | 2.53±0.11 | 2.56±0.13 | ns |
| Omental Fat (g) | 3.88±0.30 | 2.88±0.18 | ns | 2.53±0.11 | 2.56±0.13 | ns |
| Total kidney (g) | 150±8 | 117±3 | *** | 1.82±0.07 | 1.76±0.03 | ns |
| ^†^Total adrenal (g) | 5.39±0.80 | 3.64±0.45 | ns | 66.4±11.2 | 54.7±6.7 | ns |
| Liver (g) | 697±89 | 627±20 | ns | 8.55±1.11 | 9.42±0.21 | ns |
| Pancreas (g) | 70.7±5.8 | 61.8±4.1 | * | 0.86±0.07 | 0.93±0.06 | ns |
| Spleen (g) | 121±9 | 100±9 | ns | 1.48±0.12 | 1.49±0.12 | ns |
| Lungs (g) | 594±26 | 552±43 | ns | 7.25±0.39 | 8.28±0.59 | ns |
| Heart (g) | 316±9 | 271±8 | *** | 3.86±0.14 | 4.07±0.10 | ns |
| Septum (mm) | 18.3±0.99 | 14.4±0.36 | *** | - | - | - |
| Left Ventricle (mm) | 17.6±0.89 | 15.6±1.0 | ns | - | - | - |
| Right Ventricle (mm) | 7.42±0.52 | 6.71±0.60 | ns | - | - | - |

Body and organ wet weights of male (n=11) and female (n=9) offspring at *post mortem*. Data are Grand Means±SEM. Statistics are *, **, ***, P<0.05, P<0.01 or P<0.001 respectively for male *vs.* female. ns, not significant. ^†^, relative adrenal and pituitary weight expressed as (g.kg^-1^) ×10^3^.
