# Supplementary figures and images for: Adult-Onset Obesity Reveals Prenatal Programming of Glucose-Insulin Sensitivity in Male Sheep Nutrient Restricted during Late Gestation
Source: PLoS One. 2009 Oct 14;4(10):e7393. doi: 10.1371/journal.pone.0007393 (PMC2756957; doi:10.1371/journal.pone.0007393)

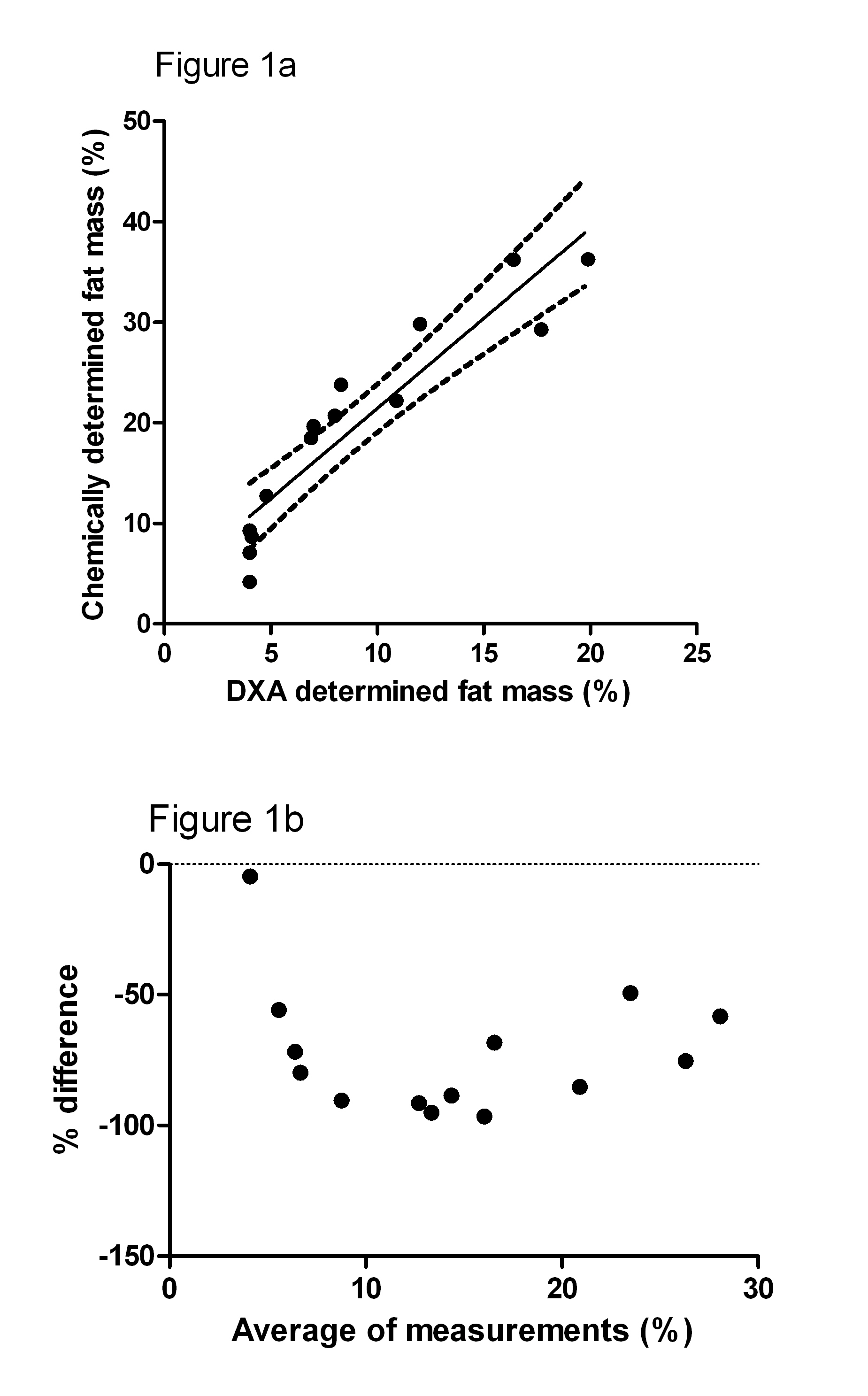

Supplement: Figure S1 — A) Validation of DXA-determined vs. chemically determined fat mass in sheep with B) a representative Bland-Altman plot. A) 14 sheep of differing body composition as determined by DXA were euthanised and body composition determined by chemical analysis (see Methods for details). The two methods were significantly correlated (P<0.001, Pearson correlation; spline with 95% CI shown) with an equation for the line of y = 1.79x+3.51. B) The Bland Altman plot illustrates the lower limit of detection for DXA in the sheep (4–5% fat) and that DXA vs. chemical analysis estimates ∼75% less whole body fat. (1.11 MB TIF) [file pone.0007393.s004.tif]

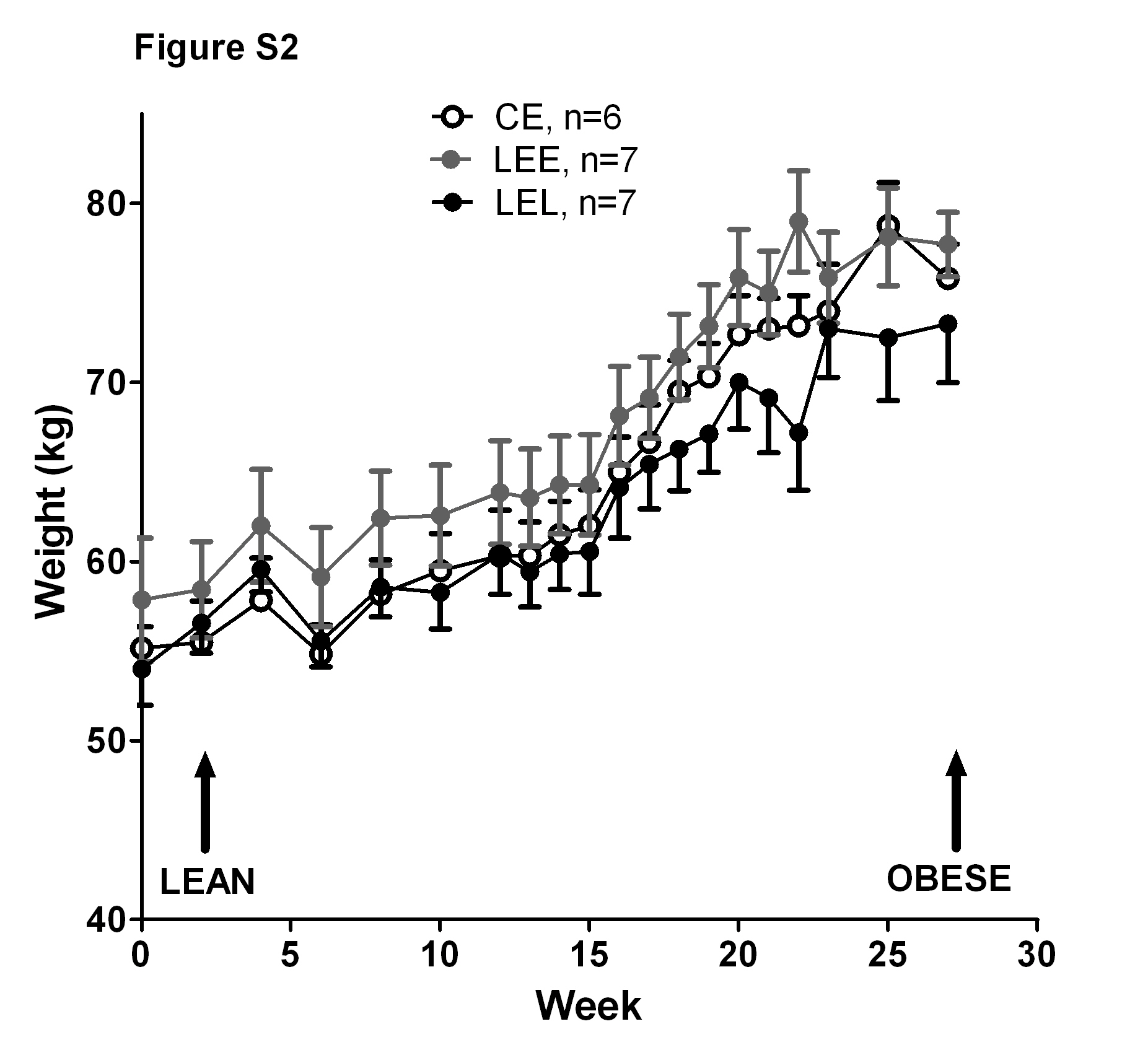

Supplement: Figure S2 — Weight gain in sheep reared in an ‘obesogenic’ environment. Data are Means±SEM. Sheep were group housed in a barn and fed at 1.5M for a period of 6–7 months to achieve a specified weight gain (see Methods). Baseline studies were conducted when animals were designated as ‘lean’ and were repeated when ‘obese’. There were no differences in weight gain between treatment groups. (0.89 MB TIF) [file pone.0007393.s005.tif]

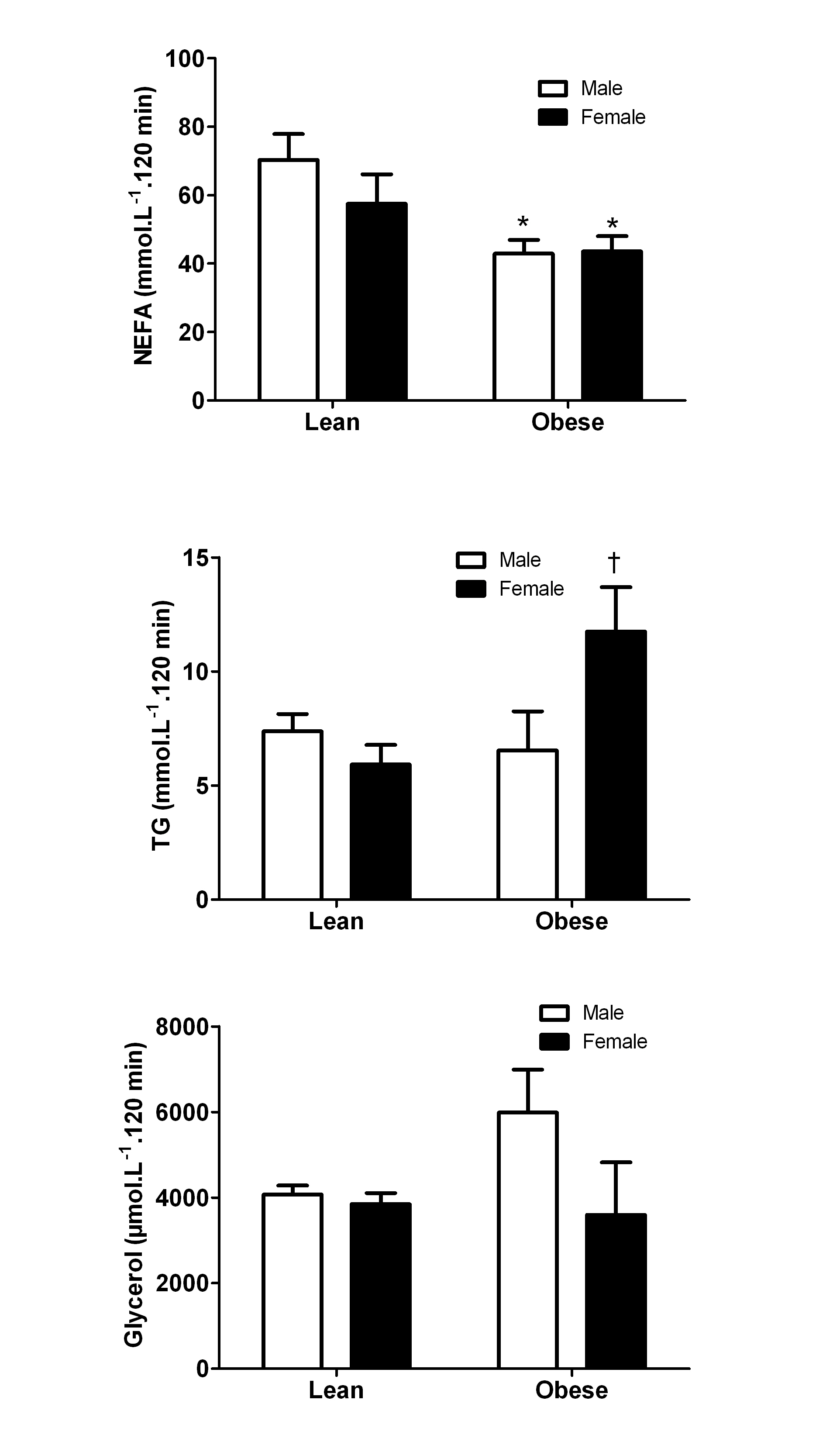

Supplement: Figure S3 — The lipid metabolite response to a glucose tolerance test in male and female lean and obese sheep. Data are Mean±SEM for areas under the glucose response curve (respective AUC units). Statistics are *, P<0.05, lean vs. obese or †, for a time*gender interaction. (2.55 MB TIF) [file pone.0007393.s006.tif]

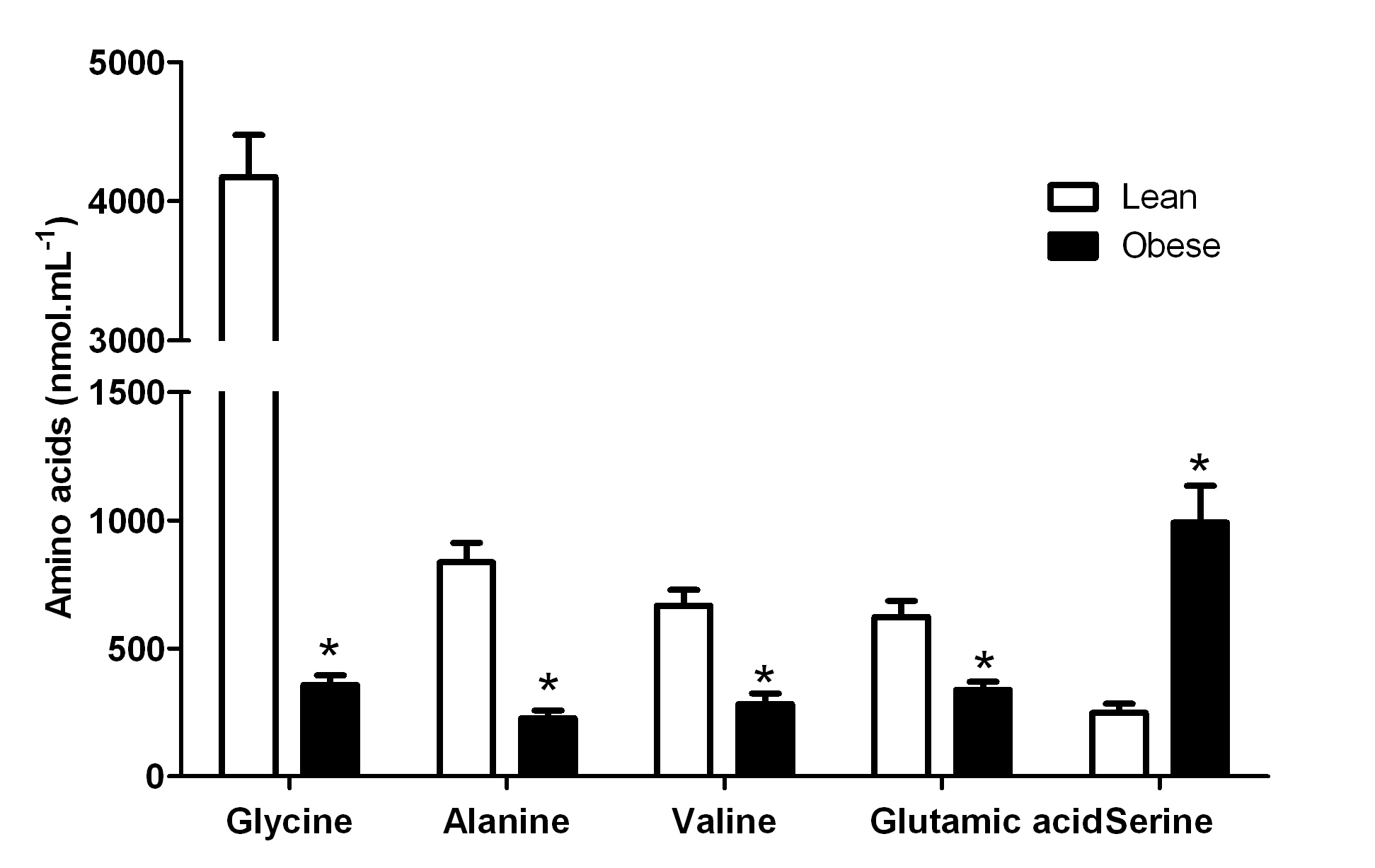

Supplement: Figure S4 — Amino acids contributing most to the variation in concentration with adult-onset obesity. Data are Mean±SEM for amino acid concentrations. Statistics are *, P<0.01, lean vs. obese. (0.81 MB TIF) [file pone.0007393.s007.tif]

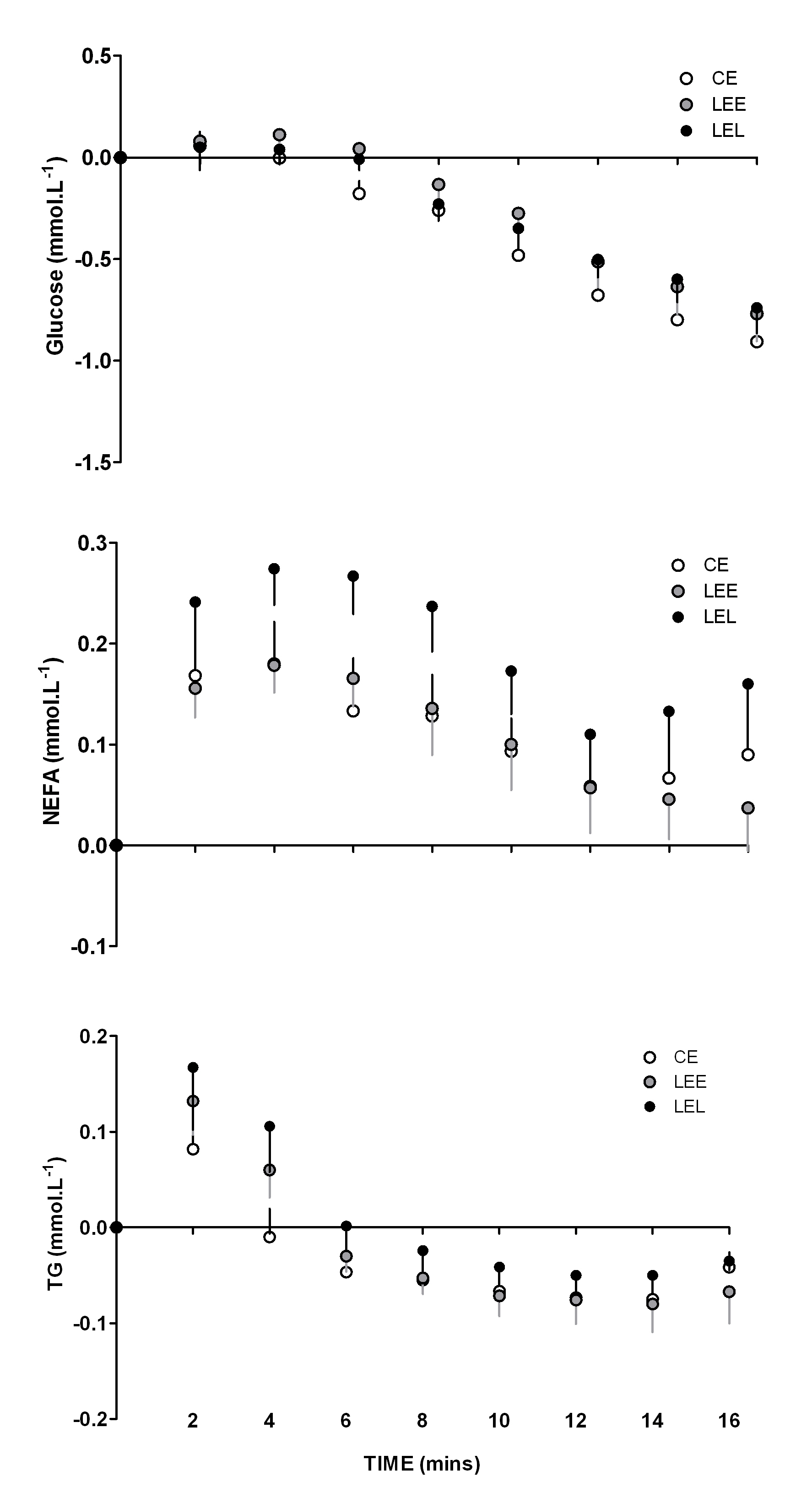

Supplement: Figure S5 — The glucose, NEFA and triglyceride response of prenatally nutrient restricted adult sheep to an insulin tolerance test when obese. Data are Mean±SEM. Insulin (0.75IU.kg-1) was injected I.V. at time zero. (0.90 MB TIF) [file pone.0007393.s008.tif]
